# Resveratrol’s Anti-Cancer Effects through the Modulation of Tumor Glucose Metabolism

**DOI:** 10.3390/cancers13020188

**Published:** 2021-01-07

**Authors:** Aranka Brockmueller, Saba Sameri, Alena Liskova, Kevin Zhai, Elizabeth Varghese, Samson Mathews Samuel, Dietrich Büsselberg, Peter Kubatka, Mehdi Shakibaei

**Affiliations:** 1 Musculoskeletal Research Group and Tumor Biology, Chair of Vegetative Anatomy, Institute of Anatomy, Faculty of Medicine, Ludwig-Maximilian-University Munich, Pettenkoferstrasse 11, D-80336 Munich, Germany; Aranka.Brockmueller@med.uni-muenchen.de; 2 Department of Molecular Medicine and Genetics, Hamadan University of Medical Sciences, 6517838678 Hamadan, Iran; saba.sameri93@gmail.com; 3 Department of Obstetrics and Gynecology, Jessenius Faculty of Medicine, Comenius University in Bratislava, 036 01 Martin, Slovakia; liskova80@uniba.sk; 4 Department of Physiology and Biophysics, Weill Cornell Medicine-Qatar, Education City, Qatar Foundation, Doha 24144, Qatar; kez4003@qatar-med.cornell.edu (K.Z.); elv2007@qatar-med.cornell.edu (E.V.); sms2016@qatar-med.cornell.edu (S.M.S.); dib2015@qatar-med.cornell.edu (D.B.); 5 Department of Medical Biology, Jessenius Faculty of Medicine, Comenius University in Bratislava, 036 01 Martin, Slovakia; peter.kubatka@uniba.sk

**Keywords:** anti-tumor action, combinatorial therapy, tumor glucose metabolism, resveratrol, warburg phenomenon

## Abstract

**Simple Summary:**

The prevention and treatment of cancer is an ongoing medical challenge. In the context of personalized medicine, the well-studied polyphenol resveratrol could complement classical tumor therapy. It may affect key processes such as inflammation, angiogenesis, proliferation, metastasis, glucose metabolism, and apoptosis in various cancers because resveratrol acts as a multi-targeting agent by modulating multiple signal transduction pathways. This review article focuses on resveratrol’s ability to modify tumor glucose metabolism and its associated therapeutic capacity. Resveratrol reduces glucose uptake and glycolysis by affecting Glut1, PFK1, HIF-1α, ROS, PDH, and the CamKKB/AMPK pathway. It also inhibits cell growth, invasion, and proliferation by targeting NF-kB, Sirt1, Sirt3, LDH, PI-3K, mTOR, PKM2, R5P, G6PD, TKT, talin, and PGAM. In addition, resveratrol induces apoptosis by targeting integrin, p53, LDH, and FAK. In conclusion, resveratrol has many potentials to intervene in tumor processes if bioavailability can be increased and this natural compound can be used selectively.

**Abstract:**

Tumor cells develop several metabolic reprogramming strategies, such as increased glucose uptake and utilization via aerobic glycolysis and fermentation of glucose to lactate; these lead to a low pH environment in which the cancer cells thrive and evade apoptosis. These characteristics of tumor cells are known as the Warburg effect. Adaptive metabolic alterations in cancer cells can be attributed to mutations in key metabolic enzymes and transcription factors. The features of the Warburg phenotype may serve as promising markers for the early detection and treatment of tumors. Besides, the glycolytic process of tumors is reversible and could represent a therapeutic target. So-called mono-target therapies are often unsafe and ineffective, and have a high prevalence of recurrence. Their success is hindered by the ability of tumor cells to simultaneously develop multiple chemoresistance pathways. Therefore, agents that modify several cellular targets, such as energy restriction to target tumor cells specifically, have therapeutic potential. Resveratrol, a natural active polyphenol found in grapes and red wine and used in many traditional medicines, is known for its ability to target multiple components of signaling pathways in tumors, leading to the suppression of cell proliferation, activation of apoptosis, and regression in tumor growth. Here, we describe current knowledge on the various mechanisms by which resveratrol modulates glucose metabolism, its potential as an imitator of caloric restriction, and its therapeutic capacity in tumors.

## 1. Introduction

Cancer cells depend heavily on their active metabolism for survival and proliferation. Most tumor cells have an adaptive and altered metabolism characterized by increased aerobic glycolysis and lactate production, leading to a significant pH gradient difference between cancerous and healthy tissues [1]. This well-known feature of cancer metabolism is referred to as the Warburg effect [2]. Indeed, in 1924, the German physiologist and Nobel laureate Otto Warburg observed that tumor cells metabolize glucose differently from healthy cells [2,3]. He reported that unlike typical mammalian tissues, most cancer cells solely “ferment” glucose to lactate, even when sufficient oxygen is present to support mitochondrial oxidative phosphorylation. Furthermore, this metabolic process enables tumor cells to produce sufficient energy to survive and proliferate despite limited resources.

Glycolysis is the primary degradation mechanism that enables mammalian cells to produce energy in the form of ATP through the oxidation of carbon bonds. The final product of glycolysis is either lactate or, after complete oxidation of glucose via the mitochondrial electron transport chain, ATP and CO_2_ [4]. In notable contrast, glucose uptake increases dramatically in tumors and other rapidly proliferating cells, even in the presence of oxygen and active mitochondria, and thus leads to lactate production. Altered carbohydrate, protein, and lipid metabolism are implicated in cancer cell proliferation and growth, apoptotic resistance, therapeutic resistance, epithelial-mesenchymal transition (EMT), metastasis, invasion, and tumor recurrence [5]. As the hyperactive glycolytic process of tumor cells is reversible, it is a potential target for treatment. Therefore, the use of drugs that mimic energy restriction for selective tumor cells that are “dependent on glycolysis” could be an auspicious anti-cancer approach. In recent years, the so-called mono-target therapies were proven unsafe, ineffective, and expensive. Notably, phytopharmaceuticals derived from fruits and vegetables exhibit promising therapeutic potential against many chronic diseases, including cancer. The plant polyphenol resveratrol (3,5,4′trihydroxy-*trans*-stilbene) was firstly isolated from the root of the white hellebore (*Veratrum grandiflorum* O. Loes) by Takaoka in 1939. Resveratrol and its biological analogs (piceatannol and pterostilbene) were found in peanuts (*Arachis* spp.), various berry species (*Vaccinium* sp.) and red wine [6]. Resveratrol is produced as a phytoalexin by plants in response to stress induced by fungi, microbes, or ultraviolet (UV) irradiation [7].

Resveratrol was first shown in 1997 to affect tumor initiation, promotion, and progression [8]. A large number of reports have since demonstrated its broad preventive and therapeutic effects against various cancer types, including gastrointestinal, breast, lung, prostate, and liver tumors. Resveratrol’s therapeutic potential is further underscored by its significant chemopreventive effects in combination with other cytostatic drugs [8].

**Figure 1:**
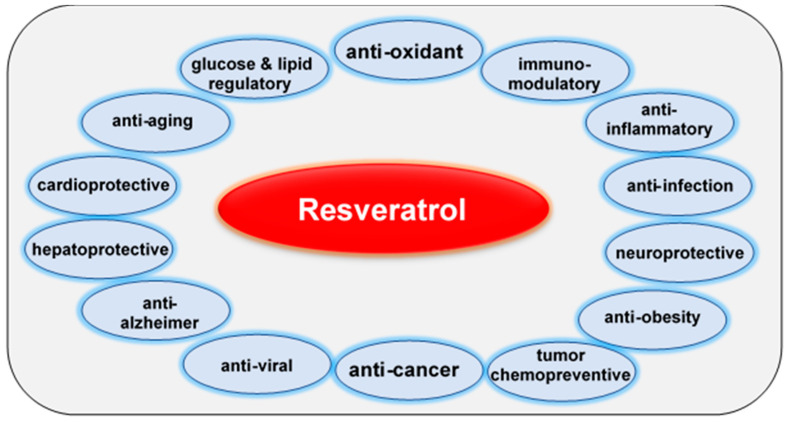
Advantages of resveratrol for patients’ health.

In traditional medicine, resveratrol has long been used as an herbal remedy. In modern medicine, resveratrol is of great interest as a “multitargeting agent” because of its anti-oxidant, anti-inflammatory, anti-obesity, anti-diabetic, anti-bacterial, anti-carcinogenic, cardio-protective, and immunomodulating properties [7,8,9,10] (Figure 1). Epidemiologically, there is an inverse association between the consumption of red wine and the incidence of cardiovascular diseases in the French population despite its high intake of saturated fats; this phenomenon is called the “French Paradox” [7,11,12,13]. Resveratrol exerts a broad spectrum of molecular effects associated with the control of cancer development. These include the reduction of glucose uptake and lactate synthesis, and consequent caloric restriction that inhibits proliferation and metastasis, and induces apoptosis [14,15,16,17,18]. Moreover, resveratrol can directly influence and modulate various metabolic enzymes and signal transduction pathways involved in oxidative glycolysis. Indeed, there is a clinical relationship between glucose resorption, diagnosis, and cancer prognosis [19,20].

This review focuses on the intracellular targets of resveratrol, and their roles in the regulation of cellular glucose metabolism and tumor growth, in the interest of improving cancer prevention and treatment.

## 2. Goal of the Review

This review deals with resveratrol’s tumor control potential, particularly its ability to suppress cancer cell glucose metabolism. We discuss the mechanisms of glucose uptake, metabolism, and degradation, as well as the molecular pathways that directly regulate tumor cell metabolism. Our review summarizes experimental studies on the anti-tumor effects of resveratrol through the modulation of glycolytic processes. Given the well-documented preclinical efficacy of resveratrol against tumor metabolism, we stress the need for targeted clinical research on the effects of resveratrol on cellular metabolic repair.

## 3. Source of the Data

Data were collected from the biomedical literature by using “resveratrol” and “cancer” or “glucose metabolism” or “glucose uptake” or “glucose transporter” or “Warburg effect” or “tumor microenvironment” or “polyphenols” or “apoptosis” as keywords or medical terms (MeSH) when searching the PubMed database.

## 4. Glucose Metabolism (Glycolysis) in Tumors and the Warburg Effect

High glucose absorption is necessary for cancer cell metabolism; this process is well regulated and involves several elements, such as growth factors [21] and interactions with the extracellular matrix [22,23]. To fulfill their glucose demands, tumor cells undergo oncogenic alterations to become independent of the processes that ordinarily regulate glucose absorption [24,25]. Essential glycolytic enzymes, glucose transporters, and transcription factors are often dysregulated during tumorigenesis [26].

In healthy cells, glucose uptake is facilitated by specific cell membrane transporters. Hexokinase phosphorylates intracellular glucose to form glucose-6-phosphate, which is subsequently converted to 3-carbon pyruvate in a process that yields NADH and ATP. In the presence of oxygen (aerobic glycolysis), healthy cells convert the intermediate pyruvate into acetyl-CoA and synthesize ATP and CO_2_ efficiently through oxidative phosphorylation (rather than glucose fermentation) [4]. In normal differentiated cells, large amounts of lactate are synthesized from pyruvate only if the oxygen supply is insufficient (anaerobic glycolysis).

**Figure 2:**
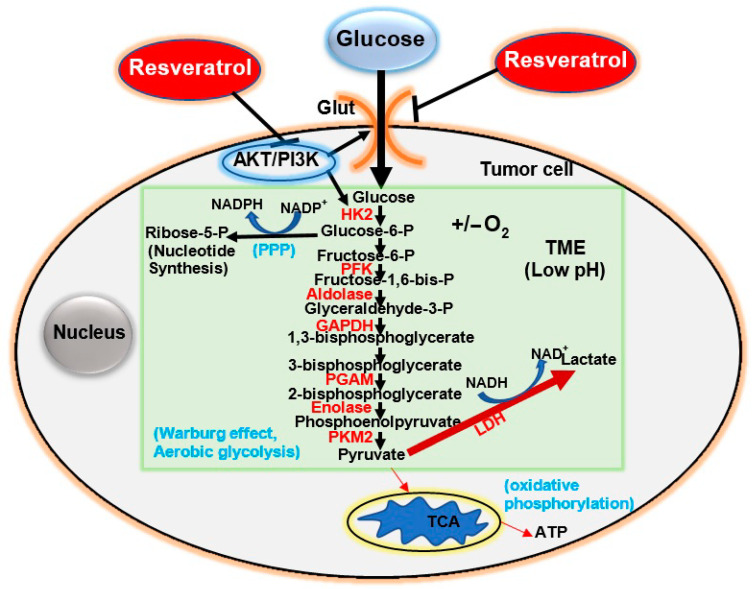
Glucose degradation and metabolism in tumor cells. The uptake of glucose by cell membrane glucose transporters (Glut) is followed by its glycolytic degradation to pyruvate. The first step is the phosphorylation of glucose by hexokinase (HK) and, under anaerobic glycolysis and aerobic glycolysis (Warburg effect, light green area), pyruvate is converted to lactate, thereby regenerating NAD+ to supply the glycolytic processes. This pathway represents an energy source for tumor cells and provides intermediates such as ribose-5-phosphate and NADPH, which are essential for cell proliferation. Pyruvate then undergoes oxidative phosphorylation in the mitochondria, which leads to the formation of ATP molecules from the tricarboxylic acid cycle (TCA). Abbreviations: ATP: adenosine triphosphate; ADP: adenosine diphosphate; NAD^+^: nicotinamide adenine dinucleotide (oxidized form); NADH, nicotinamide adenine dinucleotide (reduced form); NADPH: nicotinamide adenine dinucleotide phosphate (reduced form); TME: tumor microenvironment; TCA: tricarboxylic acid; LDH: lactate dehydrogenase.

In contrast, tumor cells are highly dependent on glucose degradation (known as fermentation) [3,27], even under aerobic conditions, to meet their high energy requirements; this is known as the Warburg Effect [28] (Figure 2, light green area). Interestingly, tumor cells can activate both the Warburg effect and mitochondrial oxidative phosphorylation simultaneously [29]. Most tumor cells synthesize large amounts of lactate independent of oxygen availability, which is why their metabolism is often referred to as “aerobic glycolysis”. Otto Warburg initially assumed that tumor cells have defective mitochondria and thus no aerobic respiratory chain [2]. This hypothesis was rejected by later research, as mitochondrial function is not impaired in most cancer cells [30,31,32].

After glucose is uptaken by membrane glucose transporters (Glut) that are overexpressed in tumor cells [33], it is converted into glucose-6-phosphate by hexokinase II (HK2). It is important to note that HK2 expression is often upregulated in malignant tumor cells [34,35,36,37], leading to increased glycolysis [38,39]. The next enzyme associated with aerobic glycolysis is phosphofructokinase (PFK), which catalyzes the phosphorylation of fructose-6-phosphate into fructose-1,6-bisphosphate; PFK is upregulated in various breast tumors [40,41]. The next step of aerobic glycolysis is the conversion of fructose-1,6-bisphosphate to glyceraldehyde-3-phosphate, catalyzed by the enzyme aldolase. Notably, aldolase is overexpressed and activated in the lung’s squamous cell carcinoma [42]. Glyceraldehyde-3-phosphate-dehydrogenase (GAPDH) then converts glyceraldehyde-3-phosphate into 1,3-bisphosphoglycerate.

GAPDH overexpression is considered an essential parameter of many tumor types [43,44,45] and a potential target for the treatment of malignant tumors [46]. In addition, phosphoglycerate mutase 1 (PGAM1) catalyzes the reversible conversion of 3-phosphoglycerate and 2-phosphoglycerate during glycolysis. PGAM1 is overexpressed in various cancer tissues and plays an essential role in cancer progression and metastasis [47]. Moreover, enolase is a key glycolytic enzyme that converts 2-bisphosphate glycerate into phosphoenolpyruvate. Enolase is overexpressed in pancreatic ductal adenocarcinoma (PDAC) tissue; its expression is correlated with metastasis and poor prognosis in PDAC patients [48]. The enzyme pyruvate kinase M2 (PKM2) catalyzes the irreversible phosphoryl group transfer from phosphoenolpyruvate to pyruvate, from which ATP is formed. In fact, tumor cells often overexpress PKM2 [49,50,51,52]. Tumor cells switch to and depend on aerobic glycolysis for survival. Therefore, lactate dehydrogenase (LDH), which catalyzes the conversion of pyruvate to lactate, is the key enzyme for determining the glycolytic phenotype of tumor cells; as such, it could be utilized as a therapeutic target. In fact, LDH inhibition suppresses the progression of lymphomas and pancreatic cancer xenografts [53]. Interestingly, Shim and colleagues (1998) reported that apoptosis could be induced by glucose deficiency in tumor cells [54]; this underscores the functional and survival importance of the Warburg effect. In summary, these findings indicate that the specific suppression of critical glycolytic enzymes could be a fundamental approach to the treatment of malignant tumors.

## 5. Glycolysis in Tumor Cells and the Pentose Phosphate Pathway (PPP)

Rapidly dividing tumor cells require a large amount of energy to drive their hyperactive proliferation, and a stable and continuous supply of nucleotides for DNA synthesis. These raw materials are supplied by the PPP. Interestingly, numerous PPP enzymes are highly dysregulated in tumors. Glucose-6-phosphate-dehydrogenase (G6PDH), a critical enzyme that determines the growth rate of tumor cells, catalyzes the first step in the PPP, thus producing. Many malignant tumors exhibit elevated G6PDH expression and PPP activity [55,56,57,58]. G6PDH knockdown significantly reduced cell proliferation; therefore, the specific inhibition of G6PDH may be an effective avenue for the treatment of glycolytic tumors [59]. The next enzyme in the PPP signaling pathway, 6-phosphogluconate-dehydrogenase (6PGDH), converts 6-phosphogluconate to ribulose-5-phosphate, and reduces NADP to NADPH. 6GPDH is an essential enzyme for lung carcinogenesis, and its specific suppression could be a new method for the treatment of glycolytic lung tumors [60]. The next enzyme in the PPP that converts ribulose-5-phosphate to ribose-5-phosphate is ribulose-5-phosphate isomerase. This enzyme is linked to tumor development [60]. The tumor cells activate *de novo* nucleotide synthesis to support their rapid proliferation and require ribose-5-phosphate for this purpose. Overall, the PPP, through its regulation of glycolysis, is essential for the survival and proliferation of tumor cells. More interestingly, it can yield promising diagnostic markers for the early detection and treatment of tumors.

## 6. The Influence of the Tumor Microenvironment on the Warburg Effect

Metabolic alterations associated with increased glycolytic degradation in tumor cells are influenced by both intracellular changes and extracellular factors in the tumor microenvironment [61]. Interestingly, a low pH (due to the Warburg effect) is the most important and consistent feature of the tumor microenvironment [62]. There is a characteristic change in the pH gradient between tumor and normal tissue [1]. Shamim and co-workers demonstrated that a low pH tumor microenvironment, associated with several factors such as reduced vascularization, nutrient deprivation, and hypoxia in the context of the Warburg effect, weakens tumor cells and supports successful anti-tumor therapy [63]. Fermentation is an essential metabolic pathway in tumor cells that maintain lower pH values (some as low as 6.0) due to lactic acid production and increased CO_2_ content [64]. The tumor-specific acidic milieu may be an important prerequisite for the effective development and action of many cancer drugs [65,66,67,68]. Notably, low pH in normal mammalian cells can cause inter-nucleosomal DNA fragmentation and apoptosis [69].

Moreover, specific influences from the tumor microenvironment can modulate DNA methylation, histone modifications, and miRNA expression, which in turn influence the metabolic processes of the tumor. All glycolytic proteins and enzymes are post-transcriptionally regulated by miRNAs. There is a relationship between the deregulation of miRNAs (such as miRNA-150, miRNA-522-3p, and miRNA-10a) and Glut1 activity [70,71,72]. Furthermore, essential enzymes such as HK2 and PKM2 are upregulated during aerobic glycolysis in tumors. It has been reported that the identification of hexokinase 2 (HK2) as a direct target of miR-143, and show that reintroduction of miR-143 in the colon cancer cell line DLD-1 leads to decreased lactate secretion. They hypothesized that loss of miR-143-mediated suppression of HK2 may promote glucose metabolism in cancer cells, contributing to the shift toward aerobic glycolysis observed in many tumors [73]. In contrast, the activation of miRNA-155 is associated with the upregulation of HK2 in lung cancer cells [74]. The expression of PKM2 is downregulated in thyroid cancer cells by miRNA-148a and miRNA-326 [75], and in cervical cancer cells by miRNA-let-7a [76]; this consequently inhibits proliferation.

## 7. Resveratrol: A Multi-Targeted Agent for the Prevention and Treatment of Chronic Diseases, Including Tumors

Resveratrol demonstrates preventive and therapeutic capacities in many chronic human diseases, including cancer. It is well established that resveratrol modulates numerous components of cell signaling pathways. Furthermore, resveratrol’s metabolic [77,78,79,80,81], hepatoprotective [82], neuroprotective [83], cardioprotective [84,85,86], anti-aging [82], anti-oxidant [87,88], anti-inflammatory [82,89], anti-diabetic [82], anti-tumor [90], cancer chemopreventive, and anti-mutagenic activities [8] have been demonstrated in recent years (Figure 1). These beneficial properties underscore its applicability in the treatment of various diseases.

Preventing and treating tumors is an ongoing medical challenge. Mono-target therapies are insufficient because they cannot meet the challenges posed by the complex pro-inflammatory tumor microenvironment, which includes numerous interactions, crosstalk’s, and regulatory mechanisms. Therefore, the identification of novel multi-targeting agents is necessary to affect both tumor cells and the multicellular tumor microenvironment. The multi-targeting activities of natural polyphenols were extensively investigated over the past twenty years. In this review, we focus on the well-studied polyphenol resveratrol. Resveratrol’s strength lies in its ability to influence several vital stages of cancer, namely tumor initiation and progression; it also exerts chemopreventive effects [8]. Specifically, it acts as a pluri-targeting agent by modulating signal transduction pathways that affect cell cycle progression, inflammation, proliferation, apoptosis, metastasis, and angiogenesis (Figure 1) in a wide range of cancer types (Table 1).

### 7.1. Resveratrol, Inflammation, and Tumors

Inflammation plays an essential and fundamental role in the development of chronic diseases. Resveratrol exerts anti-inflammatory effects through its influence on various inflammatory signaling cascades. Inflammation is a physiological response aimed to re-establish homeostasis after tissue damage caused by exogenous or endogenous factors [113]. During the inflammatory response, metabolism shifts from anabolism to catabolism ranging from the determination of the activity of adenosine monophosphate (AMP) and nicotinamide adenine dinucleotide (NAD^+^) by AMP-activated protein kinase (AMPK) and sirtuins. Thus, AMPK signaling and sirtuins functionally couple inflammation and metabolism with gene expression and transcription factors [114].

In addition, the evolutionarily conserved pro-inflammatory transcription factor nuclear factor kappa-light-chain-enhancer of activated B-cells (NF-κB) is activated by a variety of stimuli, including inflammatory cytokines and growth factors, and is significantly upregulated in many cancer cells [115,116]. NF-κB activation and NF-κB-promoting gene products are involved in tumor cell survival, proliferation, and invasion [117]. The resveratrol-Sirt1 signaling pathway significantly downregulates cancer cell migration, viability, clonogenicity, and growth by suppressing NF-κB phosphorylation [118,119,120,121], underlining the maintenance of homeostasis provides the energy metabolism balance with the inflammatory reaction [122]. Resveratrol activates the target subcellular histone deacetylase Sirt1 in various human tissues, including tumors [123,124].

### 7.2. Resveratrol and Tumors

Resveratrol exerts preventive and therapeutic effects on tumors through various mechanisms, such as the modulation of signal transduction cascades and tumor metabolism at different stages of tumor development; it can thereby affect cell proliferation, cell division, apoptosis, inflammation, angiogenesis, and metastasis (Figure 1). Resveratrol inhibits proliferation and migration and induces apoptosis by modulating glucose metabolism in various cancer types, including breast, lung, colorectal, prostate, ovarian leukemia, liver, and pancreatic cancers (Table 1). This occurs either via the caspase-3-, 8-, 9-dependent signaling pathways (receptor-mediated/type I and mitochondrial/type II) or the selective reduction of glucose uptake, transport, and metabolism through the modulation of glycolysis and induction of metabolic reprogramming [14,15,16,17,18,93,96,97,98,99,100,102,103,110,125,126,127,128,129,130,131,132,133,134,135]. Furthermore, resveratrol modulates the glucose metabolism of tumor cells switching from aerobic glycolysis (the Warburg effect; producing ATP and NADPH) to mitochondrial oxidative phosphorylation (Table 1).

**Figure 3:**
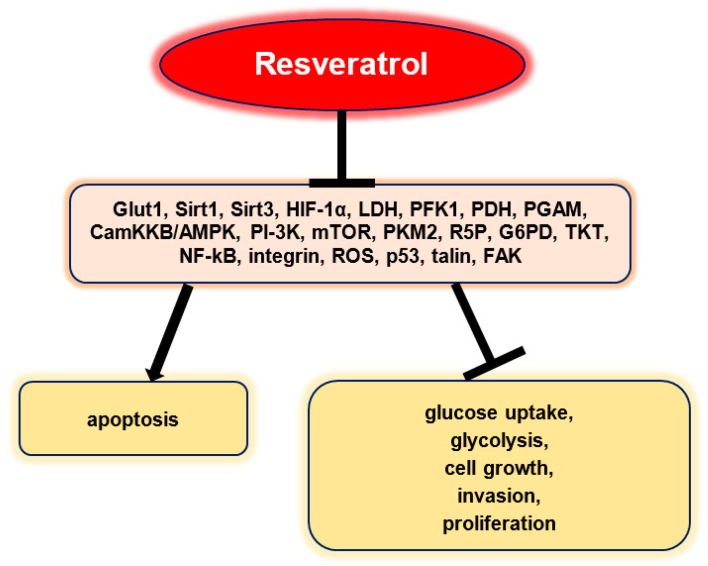
Resveratrol’s anti-cancer effects through modulation of tumor glucose metabolism.

Resveratrol is a phytochemical agent that can be used as a multi-targeted drug to supplement chemotherapy. To avoid treatment errors, co-treatment with a non-toxic, dietary, natural cancer drug that can chemosensitize and treat resistant tumors has potential [136]. Moreover, resveratrol can significantly increase the sensitivity of various cancer cells to cytostatic drugs, and improve these drugs’ action by inhibiting and/or modulating different signaling cascades, including the metabolic pathways (Figure 3) [118,137,138,139,140,141,142].

#### 7.2.1. Breast Cancer

As mentioned above, tumor cells depend primarily on glycolysis to provide the energy and intermediates required for cell growth and proliferation. The enzyme 6-phosphofructo-1-kinase (PFK) is a key glycolytic enzyme; its activity is directly associated with cellular glucose utilization. Resveratrol directly inhibits PFK activity, decreases Glut1-mediated glucose uptake, and inhibits intracellular ROS, which suppresses HIF-1α accumulation and thereby disrupts glucose metabolism and reduces the viability of breast cancer cells [15,17,98,126] (Table 1).

#### 7.2.2. Lung Cancer

Resveratrol downregulates glucose metabolism, mainly by inhibiting HK2; this is mediated by the Akt signaling pathway and leads to glycolytic suppression and ultimately apoptosis in lung cancer cells [93]. Moreover, resveratrol reduces glycolytic flux and Glut1 expression by targeting ROS-mediated HIF-1α activation in Lewis lung carcinoma tumor-bearing mice [17]. Dasari and colleagues showed that resveratrol induces autophagy in A549 lung cancer cells by upregulating glucosylceramidase beta1 (GBA1), the gene associated with Gaucher disease that codes for glucocerebrosidase, which metabolizes glucosylceramide to ceramide and glucose.

Interestingly, the expression and activity of glucocerebrosidase were significantly increased and simultaneously associated with elevated intracellular ceramide levels; both of these correlated with the occurrence of the unique death features [94]. Gu et al. further reported that resveratrol and arsenic trioxide (ATO) are involved in ROS-mediated ER stress, mitochondrial dysfunction, and apoptosis in A549 human lung adenocarcinoma cells, providing new insights into the molecular mechanisms of resveratrol-mediated ATO sensitization. This synergistic effect was combined with the upregulation of ER stress markers, including 78 kDa glucose-regulated protein (GRP-78), caspase 12, CCAAT/enhancer-binding protein-homologous protein (CHOP), cytochrome c release, and changes in Bax and Bcl-2 expression [143]. Moreover, Mollerup and colleagues demonstrated that resveratrol exerts chemopreventive effects on lung cancer through the modulation of genes involved in the metabolism of polycyclic aromatic hydrocarbons. Specifically, the inhibition of cytochrome P450 1A1 (CYP1A1) and 1B1 (CYP1B1), and upregulation of microsomal epoxide hydrolase (mEH), resulted in the modified formation of carcinogenic benzo[a]pyrene metabolites in human bronchial epithelial cells [95] (Table 1).

#### 7.2.3. Colorectal Cancer (CRC)

In colorectal cancer cells, resveratrol modulates the lipidomic activity profile, increases oxidative activity, reduces glycolysis, and decreases pentose phosphate activity; it thus reverses the Warburg effect by targeting the pyruvate dehydrogenase complex. Moreover, resveratrol improves the oxidative capacity of colorectal cancer cells via the CamKKB/AMPK signaling pathway [98], suppresses glucose metabolism and tumor growth in vitro and in vivo [97], induces apoptosis by targeting the pentose phosphate and talin-FAK signaling pathways [99], and suppresses glucose uptake by targeting ROS-mediated HIF-1α activation [17]. Furthermore, treatment of HT29 human CRC cells with resveratrol induces several ER stress markers (phosphorylation of initiation factor-2alpha (eIF-2alpha), ER stress-specific XBP1 splicing, and CHOP) and decreases glycolytic enzymes (pyruvate kinase and LDH) in Caco2 and HCT-116 cells. Simultaneously, resveratrol stimulates GRP-78, and decreases glucose uptake, Akt phosphorylation, and p-mTOR and p-p70S6K levels; these suggest the induction of ER stress. Finally, resveratrol-induced ER-stress leads to apoptosis of CRC cells [96,144,145] (Table 1).

#### 7.2.4. Prostate Cancer

Resveratrol’s anti-tumor effects (on cell growth, hydrogen peroxide production, and mitochondrial network properties) explicitly depend on the predominant oxygen (hypoxic conditions) and glucose levels; this precludes an increased dependence on oxidative phosphorylation. Resveratrol increases ROS production and the expression of the apoptotic biomarkers Bax, p53, and HIF-1α, and inhibits the anti-apoptotic protein Bcl2, thereby promoting cell death. Besides, resveratrol induces apoptosis in prostate cancer cells via the HIF-1α/ROS/p53 signaling pathway [146]. Resveratrol specifically suppresses the nuclear β-catenin protein by inhibiting HIF-1α, possibly in a proteasome-independent manner. It thereby downregulates the β-catenin-mediated transcriptional activity of androgen receptor (AR) signaling. Resveratrol thus suppresses tumor growth induces apoptosis in CRPC [147] (Table 1).

In summary, a large body of evidence shows that resveratrol inhibits the Warburg effect, reduces cancer drug resistance, and sensitizes tumor cells to chemotherapy by targeting and modulating glucose transporters and glycolytic enzymes (Table 1).

## 8. Resveratrol: Its Impact on Intracellular Molecular Signaling Targets Related to Glucose Metabolism in Tumors

Aberrant metabolism and elevated glycolytic rates in cancer cells are linked to various oncogenic processes such as proliferation, evasion of apoptosis, angiogenesis, and reprogramming of the tumor microenvironment [148]. Metabolomic studies reveal that cancers exhibit diverse metabolic phenotypes [148]. Malignant tumor cells utilize aerobic glycolysis to meet their increased glucose requirements in support of their rapid growth and proliferation; to this end, they overexpress glucose transporters (e.g., Glut1) [33,149,150,151,152]. Predominantly glycolytic tumors are characterized by the altered expression of glycolytic enzymes and transporters; therefore, these proteins represent potential targets in anti-cancer treatment [148].

Through multiple molecular targets, resveratrol suppresses growth, proliferation, and migration, and induces apoptosis, in tumor cells [153,154]. Resveratrol treatment significantly reduces glucose resorption, lactate production, and cell survival in several human ovarian cancer cell lines in a dose- and time-dependent manner [18,131,155,156]. Interestingly, resveratrol interrupts energy production by stimulating autophagy in tumor cells. Besides, resveratrol blocks glucose uptake in various tumor cells by inhibiting the cell membrane transport of Glut1 via the Akt/mTOR-dependent signaling pathway [16] (Table 2). The Akt/mTOR signaling pathway plays an essential role in the targeting of metabolism by resveratrol in tumor therapy [18,102,155]. Resveratrol also targets “classical” tumor-promoting pathways, such as PI3K/Akt, STAT3/5, and MAPK, which support glycolysis through the upregulation of glycolytic enzymes and glucose transporters [14,148,157,158,159,160] (Table 3).

Beyond the glycolytic enzymes and protein signaling molecules, glycolysis can also be regulated by miRNAs in cancers [148]. Onco-miRNAs are highly expressed in most cancers [163,164]; resveratrol administration in the Panc-1 pancreatic cancer cell line suppressed miRNA-21, leading to the inhibition of ROS-induced activation, invasion, and glycolysis [112]. PKM2, overexpressed in several cancers, was inhibited by resveratrol via the overexpression of miRNA-326 [165]. Resveratrol inhibited pancreatic cancer cell invasion and migration by suppressing ROS/miRNA-21-mediated activation and glycolysis [112]. These results provide further evidence for the association between the metabolic action of resveratrol and its anti-tumor properties (Figure 3).

## 9. Sirtuins as Major Intracellular Targets for Resveratrol in Modulating Tumor Glucose Metabolism

As previously discussed, resveratrol can alter glucose/carbohydrate metabolism in various cancers. Resveratrol modulates several signaling pathways, and thereby regulates gene expression transcription factor activity [166]. Resveratrol targets Glut1, inhibits cancer cell glucose uptake, and alters glucose utilization. Resveratrol reverses the Warburg effect and specifically targets the pyruvate dehydrogenase (PDH) complex, an important mitochondrial gatekeeper enzyme of energy metabolism, leading to increased PDH activity, inhibiting HK and PFK, and downregulating PKM2 activity [98]. Thus, it suppresses cancer cell proliferation, viability, growth, invasion, EMT, metastasis, and angiogenesis, and activates apoptotic cell death, thereby overcoming multi-drug and radioresistance [15,16,17,18,33,98,167]. One of the most important metabolic regulatory pathways stimulated by resveratrol is the molecular signaling pathway dependent on sirtuins (histone deacetylases) [168]. Resveratrol upregulates sirtuin 1 (Sirt1); Sirt1-dependent extensions of the lifespan were initially reported in yeast, worms, and flies [169,170]. Therefore, sirtuins may represent major intracellular targets for resveratrol-mediated modulation of glucose/carbohydrate metabolism in tumors. The seven highly conserved mammalian sirtuin (Sirt1-7) proteins (homologous to the yeast Silent Information Regulator 2; Sir2) are primarily a family of NAD^+^-dependent histone deacetylases. They modulate various cellular functions, including metabolism, longevity, energy homeostasis, mitochondrial function, and biogenesis, in physiological and pathological conditions [171,172,173]. The SIRTs, although localized in the nucleus (Sirt1, Sirt6, and Sirt7), cytoplasm (Sirt2), and mitochondria (Sirt3, Sirt4, and Sirt5) can reportedly translocate depending on various conditions related to the cell cycle, tissue type, developmental stage, stress, and metabolic status; this suggests that SIRT localization can regulate several metabolic pathways [174] (Table 4).

Resveratrol is a specific activator of Sirt1. Resveratrol promotes the deacetylation of many metabolic transcriptional regulators via Sirt1 in vivo [175]; this is associated with diabetes treatment [176,177], apoptosis [178], inflammation, and neuroprotection [179]. Besides, Sirt1 has significant effects on caloric restriction and life extension in cells [180,181]. Interestingly, several intracellular metabolic pathways modulated by Sirt1 are also altered during tumor development. It should be emphasized that Sirt1 can control many different oncogenic proteins and drugs and thus many cellular metabolic problems [182], such as the proliferator-activated receptor-gamma-coactivator-1 (PGC-1). Indeed, PGC-1 is activated after deacetylation by Sirt1 and regulates mitochondrial gene expression [183,184]. Sirt1 also targets other transcription factors, such as NF-κB [120], PTP1B [185] and the FOXO (Forkhead O box) family [186]. FOXO1 regulates insulin secretion, insulin resistance, and insulin signaling pathways; in turn, it can inhibit cellular glucose uptake and metabolism [185]. Furthermore, Sirt1 activation regulates the expression of many genes that control metabolism, such as pyruvate dehydrogenase lipoamide kinase 4 (PDK4) and PDH. Notably, resveratrol modulates metabolism via the Sirt1-FOXO1 signaling pathway [187].

The anti-aging effects of resveratrol could be mediated through its anti-oxidant, anti-cyclooxygenase, and anti-free radical activities, its effect on the cell cycle in vitro and in vivo, and its stimulation of Sirt1 [169,170,188,189]. Resveratrol treatment mimics the protective effect of caloric restriction against cancer by inducting Sirt1 [160,190]. In estrogen receptor-positive breast cancer cells, resveratrol elevated NAD^+^/NADH, subsequently activated Sirt1, and in turn activated the AMP-activated kinase (AMPK), a key sensor of cellular energy levels [160]. AMPK activation further inhibits the mTOR pathway and protein translation by inhibiting 4E-BP1, thereby inhibiting cancer cell proliferation [160]. Resveratrol treatment in human NSCLC cell lines upregulated Sirt1; this correlated with the loss of NF-κB function and gene expression, and rendered the cells susceptible to TNFα-induced apoptosis [191]. Resveratrol treatment mediated a dose-dependent increase in micro-RNA (miRNA-27b) correlated with the Sirt1-dependent improvement in mitochondrial function in C2C12 myoblasts and skeletal muscle [192]. Sirt6, a key regulator of glucose homeostasis and modulator of Glut1, aldolase, PDK1, and PFK1, was induced by resveratrol in FaDu hypopharyngeal carcinoma cells [193,194]. Moreover, Sirt1 plays an essential role in epigenetic modifications of the chromatin pattern and DNA repair by deacetylation [173,195]. In addition, Sirt1 controls the cellular stress response. Therefore, the state and activity of Sirt1 in cancer may play an essential role in cellular responses to epigenetic conditions and treatments.

In pathology, the SIRTs are implicated in cardiovascular diseases, diabetes, neurodegenerative diseases, age-related maladies, and cancer [168,196,197,198,199,200]. In cancers, SIRTs exert tumorigenic/tumor-promoting and tumor suppressor effects (Table 5) [174,196,200,201]. Hence, the application of resveratrol as a chemotherapeutic agent or metabolic modulator in cancers remains controversial and therefore, must be carefully studied. Resveratrol-mediated activation of Sirt1 signaling promoted human chondrosarcoma cell apoptosis and suppressed proliferation and invasion in two different colorectal cancer cells [120,202]. On the other hand, Sirt1/2/3 and 7 are implicated in breast cancer initiation, progression, metastasis, and multidrug resistance [203,204,205]. The apparently antagonistic effects of resveratrol depend on its dosage, pharmacokinetic properties, and bioavailability, and the cancer cell culture conditions [174,204,206].

## 10. Pharmacokinetics of Resveratrol: Challenges and Future Perspectives

Resveratrol occurs as two isomers in plants: *trans*- and *cis*-resveratrol [156]. The *trans*-isomer predominates in Nature [345]; it is biologically more active and more frequently studied [346] due to its higher stability [347].

Although resveratrol is extensively studied in preclinical research, its mechanisms of action under different conditions and at different doses remain elusive, as many effects demonstrated in vitro cannot be reproduced in vivo [348]. Several factors contribute to this non-reproducibility and restrict resveratrol’s clinical applicability [349]. One such factor is the compound’s pharmacokinetic profile [348]. Moreover, resveratrol has low systemic bioavailability, which may reduce its efficacy [156,350]. The bioavailability of orally administered resveratrol is less than 1% due to its rapid and extensive metabolism in the intestine and liver [345,346].

### 10.1. Absorption

Resveratrol’s appreciable solubility in alcohols and low solubility in water affects its absorption [347]. 75% of resveratrol is absorbed at the gastrointestinal level after oral administration in humans [345,346]. In the intestine, resveratrol is absorbed through passive diffusion or forms complexes with membrane transporters, such as integrins. Within the systemic circulation, resveratrol can be found in its free form or as its metabolites (glucuronide, sulfate). Free resveratrol can bind to lipoproteins or albumin; this facilitates its membrane transport and entry into cells via lipoprotein and albumin receptors [347]. The hydroxyl groups in resveratrol’s structure also enable it to associate with proteins and other nutrients. Therefore, resveratrol complexes retain their solubility and can be absorbed in the small intestine [347]. However, plasma resveratrol concentrations are not affected by protein content [351].

### 10.2. Metabolism

Resveratrol metabolism occurs via two main pathways [352]. First, the UDP-glucuronosyltransferase (UGT) enzyme family mediates the glucuronidation of resveratrol by catalyzing its conjugation to a glucuronic acid residue (either at the 3 or 4′-hydroxyl group); this alters its biological properties and facilitates its elimination from the body [352]. Critical glucuronide conjugates of resveratrol include resveratrol-4′-O-glucuronide and resveratrol 3-O-glucuronide [156,350,353]. However, the human liver microsomes contain high concentrations of UGT enzymes that preferentially form resveratrol-3-O-glucuronide [352].

### 10.3. Bioavailability and Tissue Distribution

The bioavailability and tissue distribution of resveratrol are limited. Nevertheless, resveratrol is effective in vivo; this can be explained by the reconversion of resveratrol metabolites into resveratrol in target tissues [347]. The deconjugation of enzymes such as β-glucuronidase and sulfatase and specific tissue accumulation may enhance resveratrol’s efficacy at target sites. After the stable sulfate-conjugated form of resveratrol is delivered to the target tissue, the starting compound can be regenerated to produce beneficial effects in vivo [346]. The enterohepatic recirculation of resveratrol metabolites may also explain resveratrol’s efficacy despite its low bioavailability and rapid metabolism [347].

Furthermore, there is little information on the biological activity of resveratrol metabolites [352], and preclinical research suggests that the bioactivity of glucuronated and sulfated resveratrol metabolites is weaker than that of their parent form [354]. The bioactivity of sulfated conjugates appears to decrease with increasing degrees of sulfation [352]. To the contrary, resveratrol’s beneficial effects can also be associated with its metabolites [347], as they have strong pharmacological activities [355,356]. In addition to its rapid metabolism, 75% of all resveratrol consumed is rapidly excreted. The remaining resveratrol is metabolized, and the maximum observed concentration of free resveratrol is between 1.7% and 1.9% of the initial level [357].

Interestingly, after ingestion, higher concentrations of resveratrol and its metabolites were observed in the right side of the colon than the left side [358]. Lee et al. determined that resveratrol degradation is affected by small intestinal digestion, and that in vitro human digestion decreases resveratrol’s free radical scavenging activity [359]. Nevertheless, a daily dose of 0.5 or 1 g of resveratrol produced sufficient concentrations for anti-carcinogenic effects in the human gastrointestinal tract [358].

### 10.4. Improving the Biological Effectiveness of Resveratrol

The pharmacokinetics of *trans*-resveratrol can be affected by administration routes, dosages, and treatment regimens [352]; the plasma concentration of resveratrol is associated with the ingested dose [353]. Orally administered resveratrol, in the form of 500 mg tablets, was well absorbed; the plasma concentrations of *trans*-resveratrol and its metabolites were within the reported ranges of in vitro efficacy [346]. Moreover, after repeated high dose administration in healthy volunteers, a micromolar concentration of resveratrol and much higher concentrations of its glucuronide and sulfate conjugates were observed in the plasma [360]. However, the plasma concentration of *trans*-resveratrol remained low despite high doses and a short dosing interval; nevertheless, *trans*-resveratrol’s pharmacokinetics revealed circadian variations, with higher bioavailability after morning administration [361].

As detailed in Table 6, various approaches to improving resveratrol’s biological efficacy, such as self-emulsifying drug delivery systems [362], liquid micellar formulations [363], layer-by-layer nano formulations [364], oat protein-shellac nanoparticles [365], casein nanoparticles [366], and nanocrystals [367], have yielded promising results. Selective organ targeting is also possible with resveratrol-loaded glycyrrhizic acid-conjugated human serum albumin nanoparticles (targeting the liver) [368] and *trans*-resveratrol-loaded mixed micelles (targeting the brain) [369].

Besides, the combination of resveratrol with other compounds could improve its bioavailability. Piperine, an alkaloid derived from black pepper, can inhibit glucuronidation; it may therefore increase resveratrol’s bioavailability by slowing its metabolism. As shown by Johnson et al., piperine may improve resveratrol’s bioavailability in mice [371]. Furthermore, De Santi et al. showed that quercetin could inhibit sulfotransferase 1A1 (SULT1A1) enzyme activity and thereby decrease the sulfate conjugate of resveratrol. However, this did not significantly increase resveratrol’s bioavailability [372]. Various synthetic resveratrol derivatives, such as hydroxylated, methoxylated, and halogenated derivatives, have also received attention due to their improved pharmacokinetics and biological activity compared to resveratrol [373,374].

## 11. Clinical Trials with Resveratrol

Promising preclinical findings on resveratrol’s anti-cancer effects have led to investigations into its clinical effects [375]. A study conducted on forty-two healthy volunteers demonstrated resveratrol’s capability to modulate enzymes involved in carcinogen activation and detoxification and thereby prevent carcinogenesis [376]. Resveratrol also exerted chemopreventive effects in women at increased risk of breast cancer by decreasing the methylation of the tumor suppressor *RASSF-1α* [377]. Similarly, in postmenopausal women with high BMI (BMI  ≥  25 kg/m^2^), resveratrol exerted favorable effects on hormone-related breast cancer risk factors (sex steroid hormone-binding globulin and estrogen metabolites) [378]. However, clinical evidence on the metabolic effects of resveratrol remains scarce. An in-depth search of clinical trial records yielded only indirect evidence of resveratrol’s possible effects on tumor metabolism. High concentrations of insulin-like growth factor-1 (IGF-1) and insulin promote carcinogenesis and early tumor growth through anti-apoptotic signaling and PI3K-Akt-mTORC1-mediated metabolic reprogramming. Diabetes and obesity are mainly associated with an increased risk of cancer with the Warburg phenotype [379]. A Warburg-like effect with elevated glycolysis is also present in tumors characterized by chronic treatment with insulin analogs with high affinity for the IGF-1 receptor (IGF1 and X10) [380].

Repeated administration of high-dose resveratrol in forty healthy volunteers reduced circulating IGF-I and IGF-binding protein-3 (IGFBP-3), suggesting that this mechanism is involved in resveratrol’s chemopreventive efficacy [360]. Resveratrol also reduced fasting insulin levels in patients with polycystic ovary syndrome [381]. The Wnt signaling pathway is associated with obesity and diabetes through its effects on cell metabolism, and may also be involved in metabolic reprogramming for cancer [382]. However, resveratrol can modulate the Wnt pathway [383]. Nevertheless, a phase I pilot study on colorectal cancer patients showed that resveratrol-containing freeze-dried grape powder did not inhibit the Wnt signaling pathway in colorectal cancer, but significantly inhibited Wnt expression in normal colon mucosal cells [384]. Importantly, resveratrol is safe and well-tolerated in numerous clinical studies [361,385]. However, resveratrol may be associated with mild to moderate gastrointestinal symptoms (at daily doses of 2.5–5 g) [360] or diarrhea (at twice-daily doses of 2 g) [386].

Targeting cancer cell metabolism with resveratrol could be a promising oncologic approach. However, further clinical research into the effects of resveratrol on tumor metabolism is necessary.

## 12. Conclusions and Outlook

The increasing incidence of cancer, as well as unfavorable prognoses in the event late-stage diagnosis and/or complications posed by treatment inefficacy and chemoresistance necessitate the identification of novel oncologic compounds. While metabolic and cellular signaling changes are relatively well-known processes in cancer development, the targeted manipulation of tumor metabolism can promote rapid progress in cancer treatment [387].

This review article summarizes the metabolic pathways and the associated major enzymes that contribute to the invasion, proliferation, and survival of tumor cells. Interestingly, since tumor cells’ altered metabolic processes are reversible, they represent promising therapeutic targets (Figure 3). Naturally occurring phytochemicals are currently attracting attention in all areas of cancer research [137,388,389,390,391,392,393,394,395,396,397,398,399,400,401,402]. Among these phytochemicals, resveratrol is one of the best-known polyphenolic compounds. The targeted manipulation of key metabolic enzymes by resveratrol could represent a useful and innovative therapeutic strategy to control tumors. Preclinical research results demonstrate the positive effects of resveratrol on cancer-associated metabolic processes [77,78,79,80,81,82,83,84,85,86,87,88,89,90].

To the contrary, the clinical anti-cancer efficacy of resveratrol through the regulation of tumor metabolism is not sufficiently investigated. Existing clinical trial results only indirectly indicate relationships between resveratrol and cancer metabolism [360,381,384]. Resveratrol’s application in living organisms is hindered by its low bioavailability, rapid metabolism, and pharmacokinetics [156,345,346,386]. Nevertheless, rapid progress is being made in new delivery systems to increase the efficacy of resveratrol [362,363,364,365,366,367,368,369]; this could contribute to the application of resveratrol in cancer treatment in the context of personalized medicine.

## Figures and Tables

**Table 1:** The influence of resveratrol on glucose transporters and glycolytic enzymes in tumors.

Type of Tumor	Tumor Cells	Mode of Action	Reference
Breast cancer	MCF-7 breast cancer cells.	Suppresses PFK activity, disrupting glucose metabolism and reducing viability in cancer cells.	[15]
	T47D cells, BALB/c-ν mice.	Suppresses cancer cell ^18^F-FDG uptake and glycolytic metabolism, reduces intracellular ROS, and downregulates HIF-1α accumulation.	[17]
	MDA-MB-231 breast cancer cells.	Induces apoptosis and inhibits growth by activating the *de novo* ceramide synthesis pathway.	[91]
	MCF-7 breast cancer cells.	Antiproliferative and cytotoxic effects by decreasing Glut1-mediated glucose uptake.	[92]
Lung cancer	Lewis lung carcinoma cells, BALB/c-ν mice.	Suppresses glucose uptake by targeting ROS-mediated HIF-1α activation.	[17]
	NSCLC, xenograft mouse model.	Impairs HK2-mediated glycolysis and inhibits anchorage-dependent and -independent growth of cells.	[93]
	Human lung carcinoma A549 cells.	Increases glucocerebrosidase expression, intracellular ceramide levels, and apoptosis.	[94]
	Human bronchial epithelial cell line BEP2D.	Inhibits the induced expression of CYP1A1 and CYP1B1; changes the formation and metabolism of carcinogenic benzo[a]pyrene metabolites.	[95]
Colon cancer (CRC)	HT-29 cells, BALB/c-ν mice.	Suppresses glucose uptake by targeting ROS-mediated HIF-1α activation.	[17]
	HCT116 and Caco2 cells.	Downregulates glucose uptake, glycolytic enzymes (PK, LDH), and VEGF; induces apoptosis.	[96]
	CT26 cells, CT26 tumor bearing mice.	Suppresses tumor growth and glucose uptake; increases apoptosis.	[97]
	Caco2 and HTC116 colon cancer cells.	Induces oxidative capacities through the CamKKB/AMPK pathway; increases PDH activity.	[98]
	HT-29 colon cancer cells.	Suppresses proliferation and induces apoptosis by targeting PPP and the talin-pFAK.	[99]
Prostate cancer	PC3 prostate cancer cells.	Inhibits growth via a metabolic shift from glucose fermentation to mitochondrial respiration.	[100]
	PC3 prostate cancer cells.	Suppresses tumor growth by interfering with glucose fermentation and thereby promoting oxidative respiration.	[101]
Ovarian cancer	SKOV3 and CaOV3 Ovarian cancer cells.	Reduces glucose uptake and lactate production via suppression of the Akt/mTOR pathway.	[18]
	Ovarian cancer cells.	Inhibits glucose uptake and induces apoptosis by impairing the Akt/Glut1 axis.	[102]
	A2780 and SKOV3 ovarian cancer cells.	Anti-proliferative, pro-apoptotic effects through the inhibition of glycolysis and targeting of the AMPK/mTOR pathway.	[103]
	Preclinical mouse model of ovarian cancer.	Inhibits glucose uptake with antineoplastic effects; suppresses tumor regrowth after therapy with cisplatin.	[104]
Leukemia	B-CLL and HCL lymphocytic leukemia.	Decreases the mitochondrial transmembrane potential, inhibits proliferation and growth, and induces apoptosis.	[105,106]
	CD95-sensitive leukemia cells, B-leukemic cells.	Promotes apoptosis by depolarizing mitochondrial membranes and activating caspase-9.	[107]
Liver cancer	HepG2 cells.	Suppresses glucose uptake via the Sirt1-dependent p-STAT3 signaling pathway.	[108]
	HCC, HepG2, Bel-7402, and SMMC-7721 cells.	Suppresses proliferation and migration via Sirt1-mediated post-translational modifications of the PI3K/Akt pathway.	[109]
Pancreatic cancer	Panc-1 human pancreatic cancer cells.	Suppresses ROS-induced hyperglycemia; inhibits the ERK and p38-MAPK signaling pathways.	[110]
	Pancreatic cancer cells.	Suppresses proliferation and glucose uptake by targeting HIF-1α.	[111]
	Pancreatic cancer cells.	Suppresses migration by downregulating ROS/miR-21-mediated activation and glycolysis in PSCs.	[112]

**Table 2:** Resveratrol suppresses glucose absorption and metabolism in tumors.

Experimental Model	Study Type	Finding	Reference
Nuclear magnetic resonance spectroscopy identified glycolysis as the primary glucose catabolic pathway.	In vitro/LY18 human diffuse large B-cell lymphoma cells.	Inhibits PI-3K signaling and glucose metabolism; induces cell cycle arrest.	[14]
Effects of resveratrol on PKM2 expression, and effects on cancer metabolism.	In vitro/HeLa, HepG2, and MCF-7 cells.	Downregulates PKM2 by inhibiting mTOR signaling, reduces glucose uptake, lactate production, and R5P.	[16]
Glycolytic metabolism, lactate production assay, hexokinase activity assay, intracellular RO assay, and ^18^F-FDG uptake.	In vitro/in vivo LLC, HT-29, T47D cells, BALB/c-v mice.	Reduces ^18^F-FDG uptake, glycolytic metabolism, intracellular ROS, and HIF-1α accumulation.	[17]
Growth inhibition assay; [^3^H]-2-deoxyglucose uptake and lactate assays.	In vitro/multiple human ovarian carcinoma cells.	Induces autophagy; inhibits glycolysis.	[18]
Glucose metabolism regulation via Glut1 modulation.	In vitro/human ovarian cancer cells.	Promotes apoptosis by impairing glucose uptake, involving Akt-regulated membrane Glut1 trafficking.	[102]
Metabolic and anti-tumor effects of resveratrol.	In vitro/in vivo CT26 colon cancer cells; tumor bearing mice.	Resveratrol-nanoparticles (NP) increase apoptosis and reduce ^18^F FDG uptake and ROS.	[97]
The effects of resveratrol on glucose metabolism.	In vitro/in vivo human ovarian cancer cells; murine xenograft model.	Inhibits glycolysis and glucose uptake by activating AMPK/mTOR; inhibits growth and metastasis.	[103]
The effects of resveratrol on glucose uptake and accumulation, and Glut1.	In vitro/HL-60 and U-937 leukemic cell lines.	Resveratrol blocks Glut1-mediated hexose uptake.	[134]
Mice were treated with cisplatin, resveratrol, or vehicle alone. The effect of resveratrol on glucose uptake was determined using micro-positron emission.	In vivo/murine xenograft model of ovarian cancer.	Inhibits glucose uptake, with antineoplastic effects; suppresses tumor regrowth after cisplatin therapy.	[135]

**Table 3:** Multiple intracellular metabolic enzymes as signaling molecule targets of resveratrol in tumors.

Molecular Signaling Target	Study Type	Finding	Reference
PI3K signaling	In vitro/B cell lymphoma.	Suppresses glucose catabolism; induces growth arrest.	[14]
PFK1	In vitro/human breast cancer cells.	Suppresses glucose uptake and tumor viability.	[15]
mTOR, PKM2, R5P	In vitro/HeLa, HepG2, and MCF-7 cells.	Suppresses glucose uptake and growth.	[16]
HIF-1α/ROS signaling	In vitro/in vivo HT-29 cells; BALB/c-ν mice.	Suppresses glycolytic metabolism.	[17]
Glut1	In vitro/in vivo HL-60, U-937, and HT-29 cells; BALB/c-ν mice.	Suppresses glucose uptake.	[17,33,134]
FAK, NF-κB, Integrin	In vitro/human CRC cells.	Induces apoptosis.	[119]
Sirt1, PDH, Glut1, NF-κB	In vitro/human CRC cells.	Suppresses glucose uptake and invasion; induces apoptosis.	[120]
Glut1, LDH, Sirt1, Sirt3	In vitro/human melanoma cells.	Suppresses proliferation; induces cell cycle arrest and apoptosis.	[127]
PDH, CamKKB/AMPK	In vitro/human CRC cells.	Induces oxidative capacities; inhibits glycolysis.	[98]
G6PD, TKT, Talin	In vitro/human CRC cells.	Suppresses proliferation and invasion.	[99]
HIF-1α/ROS/p53 signaling	In vitro/prostate cancer cells.	Induces apoptosis.	[146]
HK2	In vitro/in vivo.	Inhibits apoptosis.	[161]
PGAM	In vitro/human prostate cancer cells.	Inhibits tumor cell growth.	[162]

**Table 4:** SIRTs and role in metabolism.

Sirtuin	Metabolic Pathways Affected
SIRT1	Gluconeogenesis; Glycolysis; Insulin Synthesis and Secretion; Cholesterol and Fatty Acid Synthesis
SIRT2	Gluconeogenesis; Triglyceride Synthesis
SIRT3	Glutamine Metabolism; Ketone Body Formation; Urea Cycle; β-Oxidation of Fatty Acids
SIRT4	Glutamine, Leucine, and Carbohydrate Metabolism; β-Oxidation of Fatty Acids
SIRT5	Glycolysis; Tri-Carboxylic Acid Cycle; Ketone Body Formation
SIRT6	Gluconeogenesis; Glycolysis; β-Oxidation of Fatty Acids
SIRT7	Lipid Metabolism

**Table 5:** SIRTs and cancer: Localization, enzymatic activity, targets, and roles in various cancers.

Sirtuin	Cellular Location	Histone Targets	Enzymatic Activity	Oncogenic in Cancers	Genes/Targets as Promoter	Suppressor in Cancers	Genes/Targets as Suppressor
SIRT1	Nucleus	H1-K26Ac H3-K9Ac H4-K16Ac H4-K20Ac H3-K9Ac	Deacetylase	TC [207,208,209], CRC [210,211], Leukemia [212,213], RB [214], Glioma [215,216], BC [217,218,219,220], PC [209,221,222], NSCLC [217,223,224], GC [225,226], HCC [227,228,229,230], OC [231,232], PAC [233,234], SSCC [235], Melanoma [236]	c-MYC, Oct4, ZEB1, Nanog, Cripto, Tert, Lin28, STAT5, EGF, FOXO1, p53, Ku70, Rb, EMT pathway, Akt, KRas, PI3K, BRCA1, Survivin, DBC1	BC [237,238], NSCLC [239], HCC [209,237], OC [231,237], GC [240], PC [237], CRC [241,242], PAC [243], GB [237]	BRCA1, KRas, PI3K, Smad4/β-catenin, RCA1, Survivin
SIRT2	Cytoplasm	H3-K18Ac H3-K56Ac H4-K16Ac	Deacetylase, Demyristoylase	HCC [244,245], NB [246], PAC [246], GC, BC [247,248], NSCLC [249], RCC [250], GB [251], SSCC [235], Melanoma [252]	Slug, α-tubulin, c-MYC, Akt/GSK3β/β-catenin axis	OC [253], BC [254], NSCLC [255,256,257,258,259], CCA [260], HCC [254], PC [261], CRC [262], PAC [258,263], SSCC [264], SBCC [265]	CDK4, APC, CDC20, p53, c-MYC, PKM2, KRas
SIRT3	Mitochondria		Deacetylase	BLC [266], CRC [267,268], NSCLC [269], Leukemia [270], OSCC [270], OC [271], SSCC [235], Melanoma [272], BC [273]	p53, Akt/PTEN, Bax/Bcl2, Akt, RIP, SHMT2, GDH	PC [274,275], HCC [276,277,278,279], PDC [280], OSCC [281], OC [271], TC [282], GC [283,284,285], PAC [286], SBCC [265], BC [287,288,289]	c-MYC, PI3K/Akt, mTOR, Bax/Bcl2, p53, RIP, FOXO3A, Wnt/β-catenin, Twist, HIF-1α, SOD, IDH2, Notch1
SIRT4	Mitochondria		ADP-ribosylase	SSCC [235], BC [290]		NSCLC [291,292,293], ESCC [294], CRC [295,296,297], GC [291,295,298], HCC [299], RCC [300], OC [291,295], NB [301], BC [291,295,302]	GDH, ERK/Drp1 axis, E-cadherin, LKB1/AMPKα/mTOR Axis
SIRT5	Mitochondria		Deacetylase, Desuccinylase Deglutarylase Demalonylase	CRC [303,304], OSA [305], NSCLC [306,307], HCC [308], RCC [309], SSCC [235], Melanoma [310], BC [311]	Glut1, SHMT2, PKM2, E2F1, SDHA, Vimentin, GDH	NB [312], HCC [313], BC [311]	SOD
SIRT6	Nucleus	H3-K9Ac H3-K56Ac H3-K18Ac	Deacetylase ADP-ribosylase Demyristoylase Depalmitoylase	HCC [314], SSCC [315,316], Melanoma [317,318], NSCLC [319], TC [320], BC [321]	Bax, COX2, Akt, AMPK, Akt/PTEN, JAK2/STAT3, ERK1/2	HCC [322,323,324,325,326], CRC [327,328], ACC [329], GBM [330], NSCLC [331], OC [332], PAC [327,333], Melanoma [334], BC [335]	PKM2, PTEN/Akt, NF-κB, JAK2/STAT3, Bax, HIF-1α, c-MYC, Twist (EMT), Notch3
SIRT7	Nucleus	H3-K18Ac	Deacetylase Desuccinylase	OSA [336], OC [337], BC [338], GC [339], HCC [340], PC [341], SSCC [235]	CDC4, NF-κB, p38-MAPK, Bax/Bcl2	OSCC [342], BC [343]	SMAD4, NF-κB, p38-MAPK, TGFβ, EMT signaling

ACC = Adrenocortical Carcinoma, BC = Breast Cancer, BLC = Bladder Cancer, CC = Cervical Cancer, CCA = Cholangiocarcinoma, CRC = Colorectal Cancer, ESCC = Esophageal Squamous Cell Carcinoma, GBM = Glioblastoma, GC = Gastric Cancer, HCC = Hepatocellular Carcinoma, NB = Neuroblastoma, NSCLC = Non-Small Cell Lung Carcinoma, OC = Ovarian Cancer, OSA = Osteosarcoma, OSCC = Oral Squamous Cell Carcinoma, PAC = Pancreatic Cancer, PC = Prostate Cancer, PDC = Pancreatic Ductal Cancer, RB = Retinoblastoma, RCC = Renal Cell Carcinoma, SBCC = Skin Basal Cell Carcinoma, SSCC = Skin Squamous Cell Carcinoma, TC = Thyroid Cancer. Table adapted from [174,196,344].

**Table 6:** Recent developments in increasing the biological activities of resveratrol.

Delivery System	Study Details	Results	Reference
Self-emulsifying drug delivery systems of resveratrol.	In vitro, in vivo (rats).	Increased solubility, reduced metabolism, and improved its pharmacokinetic profile.	[362]
Micellar solubilization of resveratrol.	Twelve healthy volunteers (oral administration).	Increased oral bioavailability.	[363]
Layer-by-layer nanoparticles, resveratrol nanocores, and free resveratrol suspension.	In vivo (Wistar rats, oral administration, 20 mg/kg).	Layer-by-layer nanoparticles and resveratrol nanocores enhanced systemic exposure compared to free resveratrol.	[364]
Oat protein-shellac nanoparticle delivery system.	In vitro, in vivo (rat model).	Protected resveratrol in gastric fluid and controlled its release into the small intestine. Improved cell uptake and transport compared to free resveratrol. Increased bioavailability.	[365]
Encapsulation of resveratrol in casein nanoparticles.	In vitro, in vivo (rats).	Oral administration in rats: remained in the gut and reached intestinal epithelium. Produced high plasma levels of resveratrol (sustained for at least 8 h) and similar results for its metabolites. Oral bioavailability was 10 times higher compared to an oral solution of resveratrol.	[366]
*Trans*-resveratrol nanocrystals.	In vitro, in vivo (rats).	Increased oral bioavailability.	[367]
Resveratrol-loaded glycyrrhizic acid-conjugated human serum albumin nanoparticles.	In vivo (rats; single-dose tail vein injection).	Improved bioavailability of resveratrol. Elevated concentrations in the main organs of rats compared to pure resveratrol. Highest concentrations were observed in the liver (promising liver-targeted delivery system).	[368]
*Trans*-resveratrol-loaded mixed micelles.	In vivo (rats; intravenous administration).	Enhanced pharmacokinetic parameters. Brain targeting.	[369]
Resveratrol-bovine serum albumin nanoparticles (RES-BSANP).	In vivo (nude mice; intraperitoneal injection).	Improved dispersal and water solubility. Inhibited carcinoma growth in nude mice bearing human primary ovarian tumors.	[370]

## Abbreviations

6PGDH	6-phosphogluconate-dehydrogenase
Ach	Acetylcholine
AR	Androgen receptor
ATO	Arsenic trioxide
Bax	Bcl-2-associated X protein
Bcl-2	B-cell lymphoma-2
B-CLL	B-cell chronic lymphocytic leukemia
CamKKB/AMPK	Ca2^+^ Calmodulin kinase kinase B/AMP-activated kinase pathway
CHOP	CCAAT/enhancer-binding protein-homologous protein
CML	Chronic myelogenous leukemia
COX-2	Cyclooxygenase-2
CRC	Colorectal Cancer
CRPC	Castration-resistant prostate cancer
CYP1A1	Cytochrome P450, family 1, subfamily A, polypeptide 1
ECM	Extracellular Matrix
EMT	Epithelial-to-mesenchymal transition
FAK	Focal adhesion kinase
FOXO	Forkhead O box
G6PD	Glucose-6-phosphate-dehydrogenase
GBA1	Glucosylceramidase beta 1
GLUT1	Glucose transporter 1
GRP 78	Glucose-regulated protein 78
HBV	Hepatitis B virus
HCC	Hepatocellular carcinoma
HCL	Hairy cell leukemia
HER-2	Human epidermal growth factor receptor-2
HGF	Hepatocyte growth factor
HIF-1α	Hypoxia-inducible factor-1 alpha
HK2	Hexokinase II
LDH	Lactate dehydrogenase A
mEH	Microsomal epoxide hydrolase
MMP	Matrix-metalloprotease
mTORC1	Mechanistic target of rapamycin complex 1
NF-κB	Nuclear factor kappa-light-chain-enhancer of activated B-cells
NQO-1	NAD(P)H quinone oxidoreductase 1
NSCLC	Non-small-cell lung cancer
OXPHOS	Oxidative phosphorylation
PCCs	Prostate cancer cells
PDAC	Pancreatic ductal adenocarcinoma
PDH	Pyruvate dehydrogenase
PDK1	Pyruvate dehydrogenase kinase 1
PDK4	Pyruvate dehydrogenase lipoamide kinase 4
PFK1	6-phosphofructo-1-kinase
PGAM	Phosphoglycerate mutase
PGC-1	Proliferator-activated receptor-gamma-coactivator-1
PI3K	Phosphoinositide 3-kinase signaling
PK	Pyruvate kinase
PKM2	Pyruvate kinase M2
PPP	Pentose phosphate pathway
pRb	Retinoblastoma protein
PSA	Prostate-specific antigen
PSCs	Pancreatic stellate cells
PTP1B	Tyrosine-protein phosphatase non-receptor type 1
R5P	Ribose-5-phosphate
ROS	Reactive oxygen species
SREBP1	Sterol regulatory element binding protein 1
SULT	Human sulfotransferases
TKT	Transketolase
TCA	Tricarboxylic acid
UGT	UDP-glucuronosyltransferase
VDAC1	Voltage-dependent anion channel 1
VEGF	Vascular endothelial growth factor
WNT	Wnt signaling pathway
XBP1	X-box binding protein 1
